# Supplementary material for: Shaping Exploration: How Does the Constraint-Induced Movement Therapy Helps Patients Finding a New Movement Solution
Source: J Funct Morphol Kinesiol. 2022 Dec 22;8(1):4. doi: 10.3390/jfmk8010004 (PMC9844369; doi:10.3390/jfmk8010004)
Supplement: Supplementary file 1 [file jfmk-08-00004-s001.zip › jfmk-2085210-supplementary.pdf]

Supplementary Materials—Journal of Functional Morphology and Kinesiology

Shaping exploration: How does the Contraint-Induced Movement Therapy helps patients finding a new movement solution

Matheus M. Pacheco[1*]. Luisa F. García-Salazar[2]. Laura H. S. C. Gomes[3]. Fabiana S. Marques[4]. and Natalia D. Pereira[3]

[1] CIFI2D. Faculty of Sport. University of Porto - Portugal

[2] Rehabilitation Science Research Group. School of Medicine and Health Sciences. Universidad del Rosario – Colombia

[3] Department of Physiotherapy. Federal University of São Carlos (UFSCar) – Brazil

[4] Motor Development Group. Department of Physical Education. Federal University of Rondônia (UNIR) – Brazil

Performance Measures

Descriptive Measures

Table S1. Average clearing height (cm) of each participant per block of ten movements (B) and step height (S)

Part.	B1S1	B2S1	B3S1	B4S1	B5S1	B1S2	B2S2	B3S2	B4S2	B5S2
1	5.84	6.54	5.81	6.95	6.64	5.11	4.24	4.21	4.01	4.71
2	7.20	7.36	-	7.27	6.20	6.24	6.46	6.88	6.15	5.93
3	5.48	4.76	4.02	4.43	5.88	3.01	0.15	1.46	1.56	0.75
4	-	-	12.94	-	-	-	11.23	11.64	11.80	12.58

Table S2. Average peak foot height (cm) of each participant per block of ten movements (B) and step height (S)

Part.	B1S1	B2S1	B3S1	B4S1	B5S1	B1S2	B2S2	B3S2	B4S2	B5S2
1	49.84	50.54	49.81	50.95	50.64	54.11	53.24	53.21	54.01	53.71
2	31.20	31.36	-	31.27	30.20	32.74	32.96	33.38	32.65	32.43
3	20.48	19.76	19.02	19.43	20.88	22.51	19.65	20.96	21.06	20.25
4	-	-	30.94	-	-	-	32.23	32.64	32.80	33.58

Table S3. Average hip joint angle at peak foot height (degrees) of each participant per block of ten movements (B) and step height (S)

Part.	B1S1	B2S1	B3S1	B4S1	B5S1	B1S2	B2S2	B3S2	B4S2	B5S2
1	68.50	68.19	64.95	64.59	64.95	73.48	73.21	74.20	71.57	70.07
2	58.59	55.74	-	59.02	56.63	58	58.72	60.38	59.68	61.58
3	68.86	71.03	69.50	70.78	73.08	74.05	72.98	74.26	75.22	76.24
4	-	-	90.50	-	-	-	94.18	95.75	98.27	99.67

Table S4. Average knee joint angle at peak foot height (degrees) of each participant per block of ten movements (B) and step height (S)

Part.	B1S1	B2S1	B3S1	B4S1	B5S1	B1S2	B2S2	B3S2	B4S2	B5S2
1	-121.96	-123.38	-124.04	-124.02	-123.66	-123.13	-116.57	-112.09	-118.02	-115.35
2	-90.98	-92.76	-	-89.41	-86.78	-93.82	-93.01	-95.81	-93.68	-91.79
3	-77.04	-69.60	-74.64	-74.38	-77.60	-82.68	-71.25	-74.24	-74.08	-68.89
4	-	-	-83.31	-	-	-	-79.57	-81.94	-80.61	-86.53

Table S5. Average ankle joint angle at peak foot height (degrees) of each participant per block of ten movements (B) and step height (S)

Part.	B1S1	B2S1	B3S1	B4S1	B5S1	B1S2	B2S2	B3S2	B4S2	B5S2
1	22.98	22.71	22.45	22.39	22.53	22.36	21.36	20.11	21.34	21.73
2	25.34	25.32	-	24.79	24.44	26.33	25.59	25.31	25.64	24.92
3	15.28	12.84	14.47	12.11	11.14	14.85	13.65	11.29	11.11	11.18
4	-	-	3.130	-	-	-	1.900	1.670	2.010	1.820

Table S6. Average hip joint range of motion (degrees) of each participant per block of ten movements (B) and step height (S)

Part.	B1S1	B2S1	B3S1	B4S1	B5S1	B1S2	B2S2	B3S2	B4S2	B5S2
1	69.26	71.06	67.56	66.77	66.58	72.27	70.22	71.27	70.22	71.03
2	54.62	59.27	-	57.17	56.15	59.15	57.56	59.15	56.58	59.05
3	47.12	46.58	47.08	48.81	48.35	51.11	48.75	52.52	51.63	53.06
4	-	-	58.54	-	-	-	60.05	60.32	62.63	60.93

Table S7. Average knee joint range of motion (degrees) of each participant per block of ten movements (B) and step height (S)

Part.	B1S1	B2S1	B3S1	B4S1	B5S1	B1S2	B2S2	B3S2	B4S2	B5S2
1	109.53	112.91	110.41	112.93	109.93	110.79	107.55	107.8	109.95	111.05
2	91.01	94.44	-	92.30	90.47	97.62	94.77	97.06	92.14	92.85
3	76.17	70.91	70.89	74.20	72.40	79.86	73.54	76.50	72.77	74.57
4	-	-	71.37	-	-	-	71.71	74.88	78.33	86.45

Table S8. Average ankle joint range of motion (degrees) of each participant per block of ten movements (B) and step height (S)

Part.	B1S1	B2S1	B3S1	B4S1	B5S1	B1S2	B2S2	B3S2	B4S2	B5S2
1	21.54	19.96	20.05	20.40	19.18	21.29	19.93	22.05	22	23.23
2	28.99	25.96	-	26.02	24.17	27.61	26.72	25.34	25.14	26.29
3	21	21.84	22.05	23.12	23.76	22.56	21.67	25.70	23.40	21.84
4	-	-	11.82	-	-	-	11.50	10.17	9.750	9.400

Table S9. Average time (seconds) of each participant during a block of ten movements (B) and step height (S)

Part.	B1S1	B2S1	B3S1	B4S1	B5S1	B1S2	B2S2	B3S2	B4S2	B5S2
1	26.24	29.24	25.81	28.63	25.92	26.59	24.94	26.52	26.26	27.56
2	35.60	39.56	-	35.13	34.34	45.06	33.06	31.55	30.81	28.12
3	40.65	39.41	38.14	41.80	42.29	49.42	44.96	47.82	45.24	44.83
4	-	-	33.94	-	-	-	23.84	22.20	22.76	18.37

Linear Mixed Effect (LME) Models

Table S10. LME for clearing height as a function of blocks and step height ($R^2 = 0.93$)

Variable	Estimate	*t*-stat	DF	*p*-value
Intercept	7.92	4.37	30	< .001
Step Height	-1.75	2.49	30	.032
Blocks	0.01	0.08	30	.937
Step Height*Blocks	-0.06	0.26	30	.794
Intercept (random)	3.47[a]			

[a] The estimate of the random effect represents the standard deviation of the intercept provided differences between individuals

Table S11. LME for peak foot height as a function of blocks and step height ($R^2 = 0.99$)

Variable	Estimate	*t*-stat	DF	*p*-value
Intercept	32.99	5.78	30	< .001
Step Height	2.24	3.49	30	.002
Blocks	0.01	0.09	30	.931
Step Height*Blocks	-0.07	0.37	30	.717
Intercept (random)	11.38[a]			

[a] The estimate of the random effect represents the standard deviation of the intercept provided differences between individuals

Table S12. LME for hip joint angle at foot peak height as a function of blocks and step height ($R^2 = 0.98$)

Variable	Estimate	*t*-stat	DF	*p*-value
Intercept	71.75	10.95	30	< .001
Step Height	2.66	1.83	30	.077
Blocks	-0.11	0.33	30	.743
Step Height*Blocks	0.58	1.34	30	.191
Intercept (random)	12.92			

[a] The estimate of the random effect represents the standard deviation of the intercept provided differences between individuals

Table S13. LME for knee joint angle at foot peak height as a function of blocks and step height (R^2 = 0.97)

Variable	Estimate	*t*-stat	DF	*p*-value
Intercept	-93.25	10.55	30	< .001
Step Height	-1.40	0.48	30	.631
Blocks	0.06	0.09	30	.924
Step Height*Blocks	0.86	0.99	30	.326
Intercept (random)	17.16			

[a] The estimate of the random effect represents the standard deviation of the intercept provided differences between individuals

Table S14. LME for ankle joint angle at foot peak height as a function of blocks and step height (R^2 = 0.99)

Variable	Estimate	*t*-stat	DF	*p*-value
Intercept	17.18	3.84	30	< .001
Step Height	-0.61	0.86	30	.392
Blocks	-0.42	2.70	30	.011
Step Height*Blocks	0.01	0.07	30	.943
Intercept (random)	8.89			

[a] The estimate of the random effect represents the standard deviation of the intercept provided differences between individuals

Table S15. LME for hip joint range of motion as a function of blocks and step height (R^2 = 0.97)

Variable	Estimate	*t*-stat	DF	*p*-value
Intercept	58.11	15.78	30	< .001
Step Height	1.83	1.59	30	.123
Blocks	-0.13	0.53	30	.601
Step Height*Blocks	0.29	0.84	30	.408
Intercept (random)	7.16			

[a] The estimate of the random effect represents the standard deviation of the intercept provided differences between individuals

Table S16. LME for knee joint range of motion as a function of blocks and step height (R^2 = 0.96)

Variable	Estimate	*t*-stat	DF	*p*-value
Intercept	88.35	11.70	30	< .001
Step Height	0.57	0.23	30	.823
Blocks	-0.22	0.40	30	.693
Step Height*Blocks	0.36	0.48	30	.635
Intercept (random)	14.64			

[a] The estimate of the random effect represents the standard deviation of the intercept provided differences between individuals

Table S17. LME for ankle joint range of motion as a function of blocks and step height (R^2 = 0.94)

Variable	Estimate	*t*-stat	DF	*p*-value
Intercept	20.55	6.78	30	< .001
Step Height	-0.02	0.02	30	.983
Blocks	-0.23	1.02	30	.317
Step Height*Blocks	0.17	0.53	30	.598
Intercept (random)	5.86			

[a] The estimate of the random effect represents the standard deviation of the intercept provided differences between individuals

Table S18. LME for execution time as a function of blocks and step height (R^2 = 0.99)

Variable	Estimate	*t*-stat	DF	*p*-value
Intercept	32.91	7.78	30	< .001
Step Height	4.20	1.41	30	.170
Blocks	-0.08	0.13	30	.898
Step Height*Blocks	-1.48	1.66	30	.107
Intercept (random)	7.23			

[a] The estimate of the random effect represents the standard deviation of the intercept provided differences between individuals

Within-trial Exploration

Descriptive Tables

Table S19. Recurrence of the PCA-aRQA (%) of each participant during a block of ten movements (B) and step height (S)

Part.	B1S1	B2S1	B3S1	B4S1	B5S1	B1S2	B2S2	B3S2	B4S2	B5S2
1	2.36	1.89	1.29	1.46	1.41	1.32	1.70	2.60	1.97	2.07
2	2.08	1.80	-	1.73	1.87	1.49	2.10	1.63	1.31	1.76
3	1.75	2.08	1.59	1.88	1.54	1.92	1.67	2.96	1.95	1.79
4	-	-	1.80	-	-	-	1.84	1.92	2.25	1.89

Table S20. Recurrence of the cRQA (%) of each participant during a block of ten movements (B) and step height (S)

Part.	B1S1	B2S1	B3S1	B4S1	B5S1	B1S2	B2S2	B3S2	B4S2	B5S2
1	1.18	1.99	1.78	2.77	0.60	2.80	1.61	3.68	1.12	1.25
2	1.35	5.68	-	1.69	0.63	8.49	3.09	1.18	1.61	0.92
3	0.05	0.87	1.60	2.11	1.65	0.84	1.81	2.69	1.83	2.86
4	-	-	6.51	-	-	-	2.40	0.90	1.39	1.24

Table S21. Recurrence of the mdRQA (%) of each participant during a block of ten movements (B) and step height (S)

Part.	B1S1	B2S1	B3S1	B4S1	B5S1	B1S2	B2S2	B3S2	B4S2	B5S2
1	1.65	2.04	1.98	1.54	1.75	1.92	1.76	1.79	1.75	1.33
2	1.75	1.72	-	1.97	1.38	2.40	2.29	2.21	1.67	1.81
3	1.95	1.74	1.91	2.16	2.16	2.36	2.26	1.90	1.46	1.96
4	-	-	1.84	-	-	-	3.22	1.51	1.82	2.03

Table S22. Determinism of the PCA-aRQA (%) of each participant during a block of ten movements (B) and step height (S)

Part.	B1S1	B2S1	B3S1	B4S1	B5S1	B1S2	B2S2	B3S2	B4S2	B5S2
1	99.99	100	99.98	99.98	99.98	99.97	99.99	99.99	99.98	99.99
2	99.98	99.99	-	99.98	100	99.98	99.99	99.98	99.97	99.98
3	99.96	99.98	99.99	99.98	99.98	99.99	99.99	99.99	99.99	99.98
4	-	-	99.99	-	-	-	99.98	99.93	99.92	99.66

Table S23. Determinism of the cRQA (%) of each participant during a block of ten movements (B) and step height (S)

Part.	B1S1	B2S1	B3S1	B4S1	B5S1	B1S2	B2S2	B3S2	B4S2	B5S2
1	99.95	99.95	99.96	99.96	99.90	99.98	99.95	99.97	99.90	99.90
2	99.97	99.99	-	99.97	99.96	99.99	99.98	99.97	99.97	99.94
3	99.93	99.97	99.97	99.99	99.98	99.98	99.98	99.99	99.98	99.99
4	-	-	99.99	-	-	-	99.94	99.92	99.96	99.94

Table S24. Determinism of the mdRQA (%) of each participant during a block of ten movements (B) and step height (S)

Part.	B1S1	B2S1	B3S1	B4S1	B5S1	B1S2	B2S2	B3S2	B4S2	B5S2
1	98.65	99.17	98.77	98.94	98.86	98.67	98.72	98.85	98.91	98.33
2	99.35	99.41	-	99.19	99.10	99.68	99.30	99.28	99.31	98.91
3	99.74	99.73	99.67	99.79	99.76	99.86	99.87	99.87	99.77	99.72
4	-	-	99.31	-	-	-	99.21	98.08	98.15	98.69

Table S25. Entropy of the PCA-aRQA (bits/bin) of each participant during a block of ten movements (B) and step height (S)

Part.	B1S1	B2S1	B3S1	B4S1	B5S1	B1S2	B2S2	B3S2	B4S2	B5S2
1	4.04	4.19	3.77	3.94	3.89	4.01	4.15	4.28	4.15	3.99
2	3.99	4.10	-	4.15	4.02	3.90	4.14	3.95	3.89	3.96
3	3.62	3.88	3.99	4.04	4	4	4.08	4.29	4.24	4.29
4	-	-	4.10	-	-	-	3.90	3.59	3.57	2.96

Table S26. Entropy of the cRQA (bits/bin) of each participant during a block of ten movements (B) and step height (S)

Part.	B1S1	B2S1	B3S1	B4S1	B5S1	B1S2	B2S2	B3S2	B4S2	B5S2
1	3.51	3.45	3.61	3.50	3.19	3.78	3.48	3.86	3.24	3.34
2	3.74	4.47	-	3.70	3.37	4.65	3.95	3.54	3.87	3.37
3	3.13	3.73	4.01	4.16	4.12	4.02	4.26	4.50	4.25	4.29
4	-	-	4.61	-	-	-	3.90	3.32	3.82	3.32

Table S27. Entropy of the mdRQA (bits/bin) of each participant during a block of ten movements (B) and step height (S)

Part.	B1S1	B2S1	B3S1	B4S1	B5S1	B1S2	B2S2	B3S2	B4S2	B5S2
1	2.29	2.64	2.34	2.43	2.36	2.36	2.39	2.45	2.55	2.32
2	2.72	2.69	-	2.59	2.53	3.10	2.80	2.67	2.64	2.49
3	3.18	3.09	3.11	3.46	3.42	3.52	3.45	3.57	3.27	3.18
4	-	-	2.68	-	-	-	2.67	2.03	2.08	2.22

Table S28. LMAX of PCA-aRQA of each participant during a block of ten movements (B) and step height (S)

Part.	B1S1	B2S1	B3S1	B4S1	B5S1	B1S2	B2S2	B3S2	B4S2	B5S2
1	42.61	58.35	53.65	45.26	39.76	54.16	62.09	68.46	43.97	43.95
2	31.31	37.22	-	40.55	33.31	30.41	41.66	34.72	38.31	34.77
3	26.06	29.93	40.60	37.38	38.09	33.36	40.70	39.47	40.76	48.09
4	-	-	36.34	-	-	-	31.08	25.39	24.40	13.90

Table S29. LMAX of cRQA of each participant during a block of ten movements (B) and step height (S)

Part.	B1S1	B2S1	B3S1	B4S1	B5S1	B1S2	B2S2	B3S2	B4S2	B5S2
1	16.35	15.37	17.76	17.04	13.37	21.50	16.68	20.83	12.50	14.34
2	20.42	38.98	-	19.80	15.31	48.06	23.12	17.10	22	15.04
3	13.03	18.58	24.33	29.24	28.43	24.99	31.37	37.94	30.27	33.03
4	-	-	42.78	-	-	-	21.60	14.03	19.98	13.05

Table S30. LMAX of mdRQA of each participant during a block of ten movements (B) and step height (S)

Part.	B1S1	B2S1	B3S1	B4S1	B5S1	B1S2	B2S2	B3S2	B4S2	B5S2
1	5.80	7.82	5.93	6.39	5.90	6.15	6.32	6.49	7.15	5.97
2	7.70	7.72	-	7.02	6.65	10.50	8.30	7.44	7.20	6.57
3	11.74	10.57	11.07	15.14	14.26	15.43	14.77	17.03	12.62	11.56
4	-	-	7.540	-	-	-	7.47	4.78	5.19	5.38

Table S31. Laminarity of PCA-aRQA of each participant during a block of ten movements (B) and step height (S)

Part.	B1S1	B2S1	B3S1	B4S1	B5S1	B1S2	B2S2	B3S2	B4S2	B5S2
1	0.48	0.48	0.46	0.47	0.46	0.46	0.47	0.48	0.48	0.47
2	0.48	0.48	-	0.48	0.48	0.48	0.48	0.47	0.47	0.47
3	0.48	0.48	0.48	0.48	0.48	0.49	0.48	0.49	0.49	0.48
4	-	-	0.48	-	-	-	0.47	0.43	0.47	0.43

Table S32. Laminarity of cRQA of each participant during a block of ten movements (B) and step height (S)

Part.	B1S1	B2S1	B3S1	B4S1	B5S1	B1S2	B2S2	B3S2	B4S2	B5S2
1	0.66	0.65	0.60	0.60	0.61	0.66	0.68	0.59	0.63	0.56
2	0.64	0.59	-	0.57	0.52	0.51	0.51	0.56	0.43	0.51
3	0.91	0.52	0.57	0.61	0.59	0.44	0.51	0.44	0.46	0.42
4	-	-	0.48	-	-	-	0.42	0.32	0.48	0.49

Table S33. Laminarity of mdRQA of each participant during a block of ten movements (B) and step height (S)

Part.	B1S1	B2S1	B3S1	B4S1	B5S1	B1S2	B2S2	B3S2	B4S2	B5S2
1	0.54	0.49	0.44	0.39	0.45	0.44	0.44	0.39	0.45	0.46
2	0.53	0.49	-	0.46	0.49	0.48	0.49	0.47	0.51	0.51
3	0.38	0.44	0.46	0.47	0.52	0.55	0.45	0.40	0.50	0.52
4	-	-	0.45	-	-	-	0.42	0.48	0.45	0.40

Linear Mixed Models

Table S34. LME for recurrence from PCA-aRQA as a function of blocks and step height (R^2 = 0.16)

Variable	Estimate	*t*-stat	DF	*p*-value
Intercept	2.11	10.91	30	< .001
Step Height	-0.37	1.43	30	.164
Blocks	-0.12	1.98	30	.057
Step Height*Blocks	0.17	2.13	30	.041
Intercept (random)	0[a]			

[a] The estimate of the random effect represents the standard deviation of the intercept provided differences between individuals

Table S35. LME for recurrence from cRQA as a function of blocks and step height (R^2 = 0.10)

Variable	Estimate	*t*-stat	DF	*p*-value
Intercept	2.17	2.21	30	.035
Step Height	1.68	1.25	30	.221
Blocks	-0.05	0.15	30	.878
Step Height*Blocks	-0.49	1.22	30	.234

Intercept (random)	0[a]

[a] The estimate of the random effect represents the standard deviation of the intercept provided differences between individuals

Table S36. LME for recurrence from mdRQA as a function of blocks and step height (R^2 = 0.24)

Variable	Estimate	*t*-stat	DF	*p*-value
Intercept	1.83	10.15	30	< .001
Step Height	0.65	2.62	30	.014
Blocks	~[a]	0.03	30	.975
Step Height*Blocks	-0.17	2.24	30	.032
Intercept (random)	0[b]			

[a] The value was small up to 5th decimal case. [b] The estimate of the random effect represents the standard deviation of the intercept provided differences between individuals

Table S37. LME for determinism from PCA-aRQA as a function of blocks and step height (R^2 = 0.26)

Variable	Estimate	*t*-stat	DF	*p*-value
Intercept	99.97	3171.10	30	< .001
Step Height	0.04	0.90	30	.377
Blocks	~[a]	0.05	30	.958
Step Height*Blocks	-0.02	1.41	30	.170
Intercept (random)	0.02[b]			

[a] The value was small up to 5th decimal case. [b] The estimate of the random effect represents the standard deviation of the intercept provided differences between individuals

Table S38. LME for determinism from cRQA as a function of blocks and step height (R^2 = 0.33)

Variable	Estimate	*t*-stat	DF	*p*-value
Intercept	99.97	7057.80	30	< .001
Step Height	0.02	1.30	30	.203
Blocks	~[a]	0.30	30	.767
Step Height*Blocks	~[a]	1.51	30	.142
Intercept (random)	0.01[b]			

[a] The value was small up to 5th decimal case. [b] The estimate of the random effect represents the standard deviation of the intercept provided differences between individuals

Table S39. LME for determinism from mdRQA as a function of blocks and step height (R^2 = 0.78)

Variable	Estimate	*t*-stat	DF	*p*-value
Intercept	99.25	392.76	30	< .001
Step Height	0.15	0.76	30	.454
Blocks	0.01	0.32	30	.749
Step Height*Blocks	0.08	1.40	30	.173
Intercept (random)	0.41[a]			

[a] The estimate of the random effect represents the standard deviation of the intercept provided differences between individuals

Table S40. LME for entropy from PCA-aRQA as a function of blocks and step height (R^2 = 0.24)

Variable	Estimate	*t*-stat	DF	*p*-value
Intercept	3.88	26.12	30	< .001
Step Height	0.19	1.08	30	.291
Blocks	0.02	0.43	30	.673
Step Height*Blocks	-0.05	1.01	30	.321
Intercept (random)	0.14[a]			

[a] The estimate of the random effect represents the standard deviation of the intercept provided differences between individuals

Table S41. LME for entropy from cRQA as a function of blocks and step height (R^2 = 0.24)

Variable	Estimate	*t*-stat	DF	*p*-value
Intercept	3.73	15.87	30	< .001
Step Height	0.48	1.61	30	.117
Blocks	0.01	0.17	30	.866
Step Height*Blocks	-0.13	1.48	30	.149
Intercept (random)	0.17[a]			

[a] The estimate of the random effect represents the standard deviation of the intercept provided differences between individuals

Table S42. LME for entropy from mdRQA as a function of blocks and step height (R^2 = 0.87)

Variable	Estimate	*t*-stat	DF	*p*-value
Intercept	2.65	12.34	30	< .001
Step Height	0.33	2.42	30	.022
Blocks	0.01	0.35	30	.727
Step Height*Blocks	-0.10	2.37	30	.024
Intercept (random)	0.38[a]			

[a] The estimate of the random effect represents the standard deviation of the intercept provided differences between individuals

Table S43. LME for LMAX from PCA-aRQA as a function of blocks and step height (R^2 = 0.59)

Variable	Estimate	*t*-stat	DF	*p*-value
Intercept	34.44	5.67	30	< .001
Step Height	7.88	1.36	30	.185
Blocks	0.67	0.52	30	.605
Step Height*Blocks	-1.77	1.02	30	.317
Intercept (random)	8.60[a]			

[a] The estimate of the random effect represents the standard deviation of the intercept provided differences between individuals

Table S44. LME for LMAX from cRQA as a function of blocks and step height (R^2 = 0.09)

Variable	Estimate	*t*-stat	DF	*p*-value
Intercept	21.27	4.08	30	< .001
Step Height	9.75	1.37	30	.182
Blocks	0.26	0.17	30	.870
Step Height*Blocks	-2.84	1.33	30	.193
Intercept (random)	0[a]			

[a] The estimate of the random effect represents the standard deviation of the intercept provided differences between individuals

Table S45. LME for LMAX from mdRQA as a function of blocks and step height (R^2 = 0.87)

Variable	Estimate	*t*-stat	DF	*p*-value
Intercept	7.56	4.57	30	< .001
Step Height	3.06	2.94	30	.006
Blocks	0.19	0.82	30	.421
Step Height*Blocks	-0.83	2.64	30	.013
Intercept (random)	2.92[a]			

[a] The estimate of the random effect represents the standard deviation of the intercept provided differences between individuals

Table S46. LME for Laminarity from PCA-aRQA as a function of blocks and step height (R^2 = 0.46)

Variable	Estimate	*t*-stat	DF	*p*-value
Intercept	47.98	65.85	30	< .001
Step Height	-0.25	0.32	30	.752
Blocks	-0.18	1.04	30	.305
Step Height*Blocks	-0.02	0.09	30	.931
Intercept (random)	0.88[a]			

[a] The estimate of the random effect represents the standard deviation of the intercept provided differences between individuals

Table S47. LME for Laminarity from cRQA as a function of blocks and step height (R^2 = 0.51)

Variable	Estimate	*t*-stat	DF	*p*-value
Intercept	68.98	13.74	30	< .001
Step Height	-16.35	2.70	30	.011
Blocks	-3.23	2.43	30	.021
Step Height*Blocks	2.48	1.37	30	.181
Intercept (random)	4.65[a]			

[a] The estimate of the random effect represents the standard deviation of the intercept provided differences between individuals

Table S48. LME for Laminarity from mdRQA as a function of blocks and step height (R^2 = 0.69)

Variable	Estimate	*t*-stat	DF	*p*-value
Intercept	47.55	17.96	30	< .001
Step Height	-1.47	0.41	30	.687
Blocks	-0.28	0.35	30	.731
Step Height*Blocks	0.36	0.33	30	.743
Intercept (random)	0[a]			

[a] The estimate of the random effect represents the standard deviation of the intercept provided differences between individuals

Between-trial Exploration

Descriptive Tables

Table S49. Area of knee and hip joint values (at peak foot height) of each participant per block of ten movements (B) and step height (S)

Part.	B1S1	B2S1	B3S1	B4S1	B5S1	B1S2	B2S2	B3S2	B4S2	B5S2
1	85.03	138.45	40.23	53.11	39.15	109.57	78.96	40.02	70.03	82.99
2	64.48	128.27	-	193.77	254.48	98.39	223.94	119.47	109.3	232.26
3	202.5	207.76	213.69	163.97	207.51	468.12	115.53	311.04	281.44	158.19
4	-	-	135.49	-	-	-	60.07	52.33	30.77	159.3

Table S50. Variance along the equivalent space of knee and hip joint values (at peak foot height) of each participant per block of ten movements (B) and step height (S)

Part.	B1S1	B2S1	B3S1	B4S1	B5S1	B1S2	B2S2	B3S2	B4S2	B5S2
1	7.08	17.48	1.82	10.48	2.69	15.54	5.79	2.49	8.15	7.54
2	4.47	7.54	-	12.93	55.09	5.85	23.28	9.41	18.73	17.88
3	83.32	44.79	39.46	40.5	27.51	107.77	34.81	96.59	59.02	44.65
4	-	-	17.28	-	-	-	16.07	12.1	9.62	45.57

Table S51. Variance along the orthogonal space of knee and hip joint values (at peak foot height) of each participant per block of ten movements (B) and step height (S)

Part.	B1S1	B2S1	B3S1	B4S1	B5S1	B1S2	B2S2	B3S2	B4S2	B5S2
1	9.30	7.66	6.26	4.19	3.93	15.01	18.84	5	34.7	9.93
2	6.39	24.32	-	49.9	55.44	11.77	16.23	16.48	11.01	35.98
3	3.41	6.72	10.98	5.30	27.44	14.09	4	9.89	9.22	5.02
4	-	-	7.64	-	-	-	1.57	1.57	0.980	4.26

Table S52. Tolerance-cost of knee and hip joint values (at peak foot height) of each participant per block of ten movements (B) and step height (S)

Part.	B1S1	B2S1	B3S1	B4S1	B5S1	B1S2	B2S2	B3S2	B4S2	B5S2
1	0.44	0.22	0.43	0.42	0.11	0.72	1.22	0.17	2.34	0.78
2	0.02	0.83	-	1.78	1.30	0.36	0.10	0.62	0.28	0.72

3	0.02	0.50	1.47	0.25	3.63	0.85	0.65	0.03	1.16	1.19
4	-	-	0.34	-	-	-	0.18	0.19	0.07	0.04

Table S53. Number of jumps in the trial-to-trial change of knee and hip joint values (at peak foot height) of each participant per block of ten movements (B) and step height (S)

Part.	B1S1	B2S1	B3S1	B4S1	B5S1	B1S2	B2S2	B3S2	B4S2	B5S2
1	1	1	0	0	1	1	0	2	1	1
2	1	1	-	1	1	1	1	0	0	0
3	1	0	0	0	1	0	0	0	1	0
4	-	-	0	-	-	-	0	0	0	1

Linear Mixed Models

Table S54. LME for area of knee and hip joint values (at peak foot height) as a function of blocks and step height (R^2 = 0.44)

Variable	Estimate	*t*-stat	DF	*p*-value
Intercept	108.45	2.09	30	.045
Step Height	61.27	1.06	30	.296
Blocks	7.82	0.62	30	.542
Step Height*Blocks	-15.69	0.91	30	.370
Intercept (random)	59.54[a]			

[a] The estimate of the random effect represents the standard deviation of the intercept provided differences between individuals

Table S55. LME for variance along the equivalent space of knee and hip joint values (at peak foot height) as a function of blocks and step height (R^2 = 0.55)

Variable	Estimate	*t*-stat	DF	*p*-value
Intercept	25.36	1.76	30	.087
Step Height	7.76	0.52	30	.608
Blocks	-0.84	0.25	30	.801
Step Height*Blocks	-0.75	0.17	30	.867
Intercept (random)	18.48[a]			

[a] The estimate of the random effect represents the standard deviation of the intercept provided differences between individuals

Table S56. LME for variance along the orthogonal space of knee and hip joint values (at peak foot height) as a function of blocks and step height (R^2 = 0.42)

Variable	Estimate	*t*-stat	DF	*p*-value
Intercept	-1.41	0.20	30	.841
Step Height	9.99	1.22	30	.233
Blocks	5.20	2.88	30	.007
Step Height*Blocks	-4.25	1.73	30	.093
Intercept (random)	7.03[a]			

[a] The estimate of the random effect represents the standard deviation of the intercept provided differences between individuals

Table S57. LME for tolerance-cost of knee and hip joint values (at peak foot height) as a function of blocks and step height (R^2 = 0.19)

Variable	Estimate[a]	*t*-stat	DF	*p*-value
Intercept	-0.21	0.53	30	.600
Step Height	0.65	1.17	30	.252
Blocks	0.33	2.72	30	.011
Step Height*Blocks	-0.28	1.66	30	.107
Intercept (random)	0.67[b]			

[a] The data was multiplied by 100 for better visualization; [b]The estimate of the random effect represents the standard deviation of the intercept provided differences between individuals

Table S58. GLME for chance of jumps in the trial-to-trial change of knee and hip joint values (at peak foot height) as a function of blocks and step height (R^2 = 0.69)

Variable	Estimate	*t*-stat	DF	*p*-value
Intercept	0.80	0.61	30	.544
Step Height	-1.24	0.72	30	.480
Blocks	-0.14	0.38	30	.920
Step Height*Blocks	0.17	0.34	30	.734
Intercept (random)	1[a]			

[a] The estimate of the random effect represents the standard deviation of the intercept provided differences between individuals
